# ICU Nurses' Perceived Barriers to Effective Enteral Nutrition Practices: A Multicenter Survey Study

**DOI:** 10.2174/1874434601812010067

**Published:** 2018-05-25

**Authors:** Muhammad W. Darawad, Nedal Alfasfos, Ismael Zaki, Malek Alnajar, Sawsan Hammad, Osama A. Samarkandi

**Affiliations:** 1 School of Nursing, the University of Jordan, Amman, 11942 Jordan; 2 Faculty of Nursing, Alahliya Amman University, Amman, Jordan; 3 Prince Sultan bin Abdulaziz College for Emergency Medical Services, King Saud University, Riyadh, Saudi Arabia

**Keywords:** Enteral Nutrition, ICU, Nurses, Critically-ill, Jordan, Multidisciplinary

## Abstract

**Background:**

Critically ill patients are hypermetabolic and have increased energy requirements, making nutritional support a vital intervention. In the Intensive Care Units, enteral nutrition is based on opinions rather than evidence-based practices. Therefore, there is a need to identify the barriers to evidence based practice protocols for enteral feeding of patients in Jordanian ICUs.

**Aims:**

To explore Jordanian ICU nurses' perceived barriers for enteral nutrition that hinders them from utilizing the recommended EN guidelines.

**Methods:**

A descriptive cross-sectional design was utilized using self-administered questionnaire. A total of 131 nurses participated from different hospitals representing different healthcare sectors in Jordan.

**Results:**

The five barriers subscales' means were almost equal ranging from 4.04 (Delivery of EN to the Patient) to 4.33 (ICU Resources) (out of 7). The most important barrier was “Not enough nursing staff to deliver adequate nutrition” (M=4.80, SD=1.81, 60%), followed by “Fear of adverse events due to aggressively feeding patients” (M= 4.59, SD=1.50, 56%). Although no significant differences in the mean barrier score were revealed, minimal significant differences were revealed that were distributed among different barrier subscales.

**Conclusion:**

Participants moderately perceived barriers with more focus on insufficient resources in ICU and among healthcare providers. Such barriers are modifiable and manageable, making their identification and management crucial for optimal patient care. This study confirms that enteral nutrition is a multidisciplinary responsibility.

## 1 INTRODUCTION

Critically-ill patients are hypermetabolic and have increased energy requirements due to their illnesses; their nutritional support is a vital intervention [1, 2]. Multiple studies showed that current feeding practices failed to provide patients in intensive care units (ICU) with adequate feeding [3, 4]. More than 35% of ICU patients are malnourished [5], which increases their risk for infection and impairs wound healing [6], and leads to prolonged hospital stay, increased costs of health care, and increased morbidity and mortality [7], and increases their levels of pain [8]. For critically-ill patients, Enteral Nutrition (EN) is the most preferred method of nutritional support as it improves clinical outcomes and lowers health-related costs compared to parenteral nutrition [9, 10].

Singer *et al.* [10] recommend applying evidence-based approaches to improve patients' feeding and clinical outcomes. Such outcomes of EN are found to be better in ICUs that follow EN clinical practice guidelines [11]. Nursing care for patients with EN has a significant role in ensuring the success of EN [12]. Despite their crucial role in providing EN, nurses' practices are considered one of the factors that lead to underfeeding in critically-ill patients and subsequent malnutrition [13, 14]. Current nurses' practices of EN have been found to be based on opinions rather than on evidence-based practices, leading to variability in providing this vital care [15, 16].

Several studies showed a gap between the recommended guidelines and the actual practices at bedside [17, 18]. One of the most comprehensive studies was a cross sectional survey conducted among 383 ICU nurses from 20 European countries using a 51-item self-administered questionnaire to evaluate EN practices in the European ICUs. The results showed a variation in practices across different ICUs and discrepancies in following the European EN recommendations [16]. The same discrepancy in following the guidelines was reported among Egyptian nurses specifically regarding nasogastric tube (NGT) insertion and administration of medications [19], and among Jordanian nurses specifically regarding tube placement confirmation and assessment of GRV [20].

Many studies aimed to narrow the guideline-practice gap through multifaceted implementation strategies. These studies shared that implemented strategies only resulted in small changes in feeding practices without improvement in patient outcomes [21, 22]. Although there are well-acknowledged guidelines for EN use, there is a need to identify the barriers beyond the inappropriate practices regarding EN in the ICUs. A change in practice may be more likely a strategy for EN use, which are specifically chosen to address the identified barriers, treating ICU as an area that needs special focus [23, 24].

Barriers are factors that hinder the implementation of recommended guidelines in clinical practice and increase the gap between recommended guidelines and practices. Barriers could be related to individuals, social issues, or the organizations. To produce a change in practice, barriers should be identified in order to develop strategies to overcome these barriers. Cahill *et al.* [25] conducted a study to identify the most common barriers for effective EN. Their investigation revealed the following barriers; feeding tube not in place, delay in physicians' orders, delay in initiation of motility agents, lack of EN formula and/or feeding pumps, and delay in the initiation time of EN.

Providers' characteristics constituted a part of those barriers. For example, nurses' gender was found to be a barrier since female nurses have been recognized for their perception of responsibility toward the provision of EN more frequently than male nurses [26]. Barriers regarding EN varied across settings and times [23, 27]. The improvement of EN practices may be more likely if the strategies were specifically chosen to address these identified barriers. Limited studies were found to consider investigating the barriers of EN in ICU. Thus, this study aims to explore the perceived barriers that hinder Jordanian ICU nurses from utilizing the recommended EN guidelines. Specifically, this study aimed to answer the following questions:

1. What are the perceived barriers of administering EN among Jordanian ICU nurses?
2. Are there differences in the ICU nurses' perceived barriers of EN based on personal characteristics?

## 2 METHODOLOGY

### 2.1 Design

Using a self-administered questionnaire, a descriptive cross-sectional design was used to identify Jordanian ICU nurses' EN practices and the barriers that hinder them from utilizing effective practices of EN.

### 2.2 Setting

Jordanian hospitals are divided into four types of sectors; educational, private, public, and military [28]. Accordingly, one large hospital (that contains more than one ICU unit) from each sector was randomly selected from a list of hospitals in each sector, except for the military sector. The military hospitals do not allow outside scientists to collect data inside their institutions.

### 2.3 Sampling

A non-probability convenience sample was used to select ICU nurses who met the study eligibility criteria that included: 1) being a registered nurse, 2) have at least 3 months experience in the ICU, and 3) provides direct patient care. Nurses with administrative positions were excluded from the study. The sample size was calculated using G-power V.3 [29], at alpha level 0.05, power 80, and medium effect size 0.3 for bivariate correlation. Thus, the minimum required sample size to detect a significant correlation between variables was 84 nurses. However, a larger sample size was targeted to compensate for incomplete questionnaires.

### 2.4 Data Collection

Before starting data collection, data collectors met with ICU head nurses and asked for their permission to start data collection. Nurses who met the eligibility criteria and were interested to participate in the study were invited to complete a questionnaire after reading the cover letter and indicating an understanding the study purpose. The data collectors were available to answer any question as nurses completed the questionnaire, completion of the tool took approximately 10 to 15 minutes.

### 2.5 Ethical Considerations

The ethical approval to conduct the study was obtained from both the Scientific Research and Ethics Committee at the Faculty of Nursing-The University of Jordan and the participating hospitals before beginning data collection. Nurses were informed that participation in the study was completely voluntary and that they had the right to withdraw from the study. This also meant that nurses had right to provide incomplete information or answers to any statement in the questionnaire. A written cover letter contained information about the purpose of the study, the time needed for filling the questionnaire, and contact information of the primary investigator.

### 2.6 Instrument

Barriers to EF were explored using a questionnaire that contained 26 barriers. The respondents were asked to rate their importance as barriers in their ICU from 1 (not at all important) to 7 (very important). The questionnaire was divided into 5 subscales including the guideline recommendations and implementation strategies subscale (6 items), delivery ICU resources subscale (3 items), dietitian support subscale (4 items), delivery of enteral nutrition to the patient (7 items), and critical care provider attitudes and behaviors (6 items). The average of the scale out of 7 was used to compare between and among barrier items and the subscale. The internal reliability for the subscales and the overall instrument were acceptable, where the Cronbach's alpha for the subscales ranged from 0.84 to 0.89 (Cahill *et al.*, 2012).

Even though English language is not the native language of the participants, nurses are expected to understand written English and medical terminology since education at nursing schools and communication and documentation at hospitals is carried out in English. However, a pilot study was carried out to ascertain that the instrument items, which were written in English, were understood by the participants, and to check the feasibility of data collection process.

### 2.7 Data Analysis

Demographic data and the barriers scale were coded and entered into Statistical Package for the Social Sciences (SPSS) version 19.0. Descriptive statistics were used to answer research questions, and to describe the demographic variables and the EN barriers. Data were presented as frequencies and percentages for categorical variables while means and standard deviations were used for continuous variables. A series of independent sample t tests and analysis of variance (ANOVA) were used to describe differences in the ICU nurses' perceived barriers of EN based on their characteristics.

## 3 RESULTS

### 3.1 Description of the Sample

A total 131 nurses were initially recruited to the study. Participants were recruited from the three healthcare sectors in Jordan including private 43% (n=56), governmental 30% (n=40) and educational 27% (n=35). They were recruited from different types of ICUs including Surgical ICU (18.3%, n=24), Medical ICU (26%, n=34), Coronary ICU (26.7%, n=35), and others (29%, n=38). For the entire sample, the majority (53%, n=69) were males, and had a baccalaureate degree (84%, n=110). The sample age range was between 22 and 51 years (M= 27.3± 3.9), and their years of experience in nursing ranged between 1 and 27 years (M= 4.6±3.7). Regarding the presence of written guideline about enteral nutrition, 70 nurses (53%) reported no written guideline in their units. The majority of respondents (66%, n=86) reported that they had not received any previous education regarding EN.

### 3.2 Description of the Study Variables

#### 3.2.1 Enteral Feeding Barriers Items

The barriers questionnaire was composed of 26 items, divided into 5 barriers subscales. As seen in Table (**2**), the five subscales' means were almost equal ranging from 4.04 (Delivery of EN to the Patient) to 4.33 (ICU Resources) (out of 7). In order to describe items of barriers, each single item’s mean, standard deviation, and percent of agreement (somewhat important, important, and very important) were calculated to determine the most important and the least important barriers. The most important barrier was “*Not enough nursing staff to deliver adequate nutrition*” (M=4.80, SD=1.81, 60%) from the ICU Resources subscale, followed by “Fear of adverse events due to aggressively feeding patients” (M= 4.59, SD=1.50, 56%) from the Attitude subscale, and “*Feeds being held due to diarrhea*” (M= 4.58, SD=1.65, 57%) from the Attitude subscale. On the other hand, the least important barriers were “*Non-ICU physicians requesting patients not be fed enterally*” (M= 3.63, SD=1.76, 34%) from the Attitude subscale, preceded by “*No feeding tube in place to start feeding*” (M= 3.76, SD=1.88, 38%) from Delivery subscale, and “*Waiting for the dietitian to assess the patient*” (M= 3.85, SD=1.885, 37%) from the Dietitian Support subscale. When nurses were asked to rate the most three important barriers they ranked “*Not enough nursing staff to deliver adequate nutrition*” as number one (16%), and “*No feeding protocol in place to guide the initiation and progression of enteral nutrition*” as number two (9.2%), and “*No or not enough dietitian coverage during evenings, weekends and holidays*” as number three (11.5%). Table (**1**) provides detailed information about the reported means of all barriers.

#### 3.2.2 Differences in Nurses' Perceived Barriers of EN Based on Their Demographics

A series of independent sample t-test and ANOVA were conducted to assess differences of nurses' perceived barriers of EN based on their demographic characteristics. Analysis revealed no significant differences in the mean barrier score based on any demographic variable. At the level of the subscales, analysis revealed no significant differences based on nurses' gender, ICU type and presence of EN guidelines in the unit. Very minimal significant differences were revealed that were distributed among different barriers subscales (Table **2**). For instance, nurses in the private sector reported more barriers regarding “Delivery” than nurses in an educational hospital, and more barriers regarding “Attitudes” than nurses in both other sectors. Nurses with a baccalaureate degree reported more barriers regarding “ICU Resources” (M=4.48) than nurses with a Master’s degree (M=3.54), and nurses who reported having previous EN education reported fewer barriers regarding “ICU Resources” (M=3.81) than those who did not (M=4.61).

### 3.3 Reliability of Enteral Feeding Barriers Scale

To assess the reliability of the enteral feeding barriers scale (Total and subscale), Cronbach‘s alpha was computed. Results revealed that Cronbach‘s alpha ranged between .66 (ICU Resources subscale) to .85 (Delivery subscale), and a value of .89 for the total score, which indicates strong level of internal consistency for this scale.

## 4 DISCUSSION

This study evaluated the EN barriers perceived by Jordanian ICU nurses, and examined these barriers based on nurses’ demographics. This study came as a response to Cahill *et al.* [25] who stated that further research is required to illuminate on how demographic variance of nurses might influence the type of barriers encountered in EN administration. This study adds to the body of nursing knowledge about the EN field in Jordan; an area that has limited studies.

Understanding EN barriers is crucial to improve nursing practice and achieve optimal EN. In this study, the mean overall barrier score was 4.18, out of 7, which represents moderately perceived available barriers. The existence of barriers hinders the implementation of the recommended guidelines in clinical practice and increases the gap between recommended guidelines and practice [25, 27]. Since barriers to EN are multifactorial issues that differ from one institution to another, identifying specific barriers is of great importance.

Although the mean score of the barriers subscale was comparable, “ICU Resources” subscale scored the highest. This result was inconsistent with literature in which “the Delivery of Enteral Nutrition” and “Dietitian Support” scored the highest on subscales of barriers [25]. However, “*insufficient nursing to deliver nutrition”* scored the highest barrier, which was part of the “ICU Resources” and was similar to the highest barrier reported by Cahill *et al.* (2012). The problem of the nursing shortage in Jordan is well-documented in Jordanian nursing literature [30, 31], and it seems to affect the quality of nursing care at least in EN administration.

In “Attitude” subscale, nurses reported negative attitudes regarding nutritional care since they believed that that “*provision of adequate nutrition does not impact patient outcomes*”. This result was inconsistent with the study of Cahill *et al.* [25]. In addition, the item on the “Delivery” subscale “*In resuscitated and hemodynamically stable patients, other aspects of care still take priority*” had 44% of agreement with the sample. Knowing that the ICU environment is full of complicated tasks, high work pressure and insufficient staffing, patient feeding becomes a secondary priority compared to other tasks. Having negative attitudes regarding the importance of enteral nutrition, which puts this care in the least priority, predisposes critically-ill patients to under nutrition and malnourishment. Therefore, the availability of nutritional guidelines and protocols should be mandated since nutritional care is perceived as a low priority in critical care units. Promoting nurses' clinical decision making and autonomy with pre-set guidelines would promote their attitudes [32], and promote the provision of adequate enteral nutrition.

In this study, many factors were associated with EN guideline recommendations and implementations strategies such as unfamiliarity with guidelines, inadequacy and inaccessibility of the evidences to support the care. This result was consistent with the results of Hammad *et al.* [18], and Shahin *et al.* [19]. In the study of Cahill *et al.* [25], the absence of feeding protocols was not a barrier. Similar to the results reported by Roynette *et al.* [16], Jordanian healthcare sectors have different EN policies and guidelines, and the problem of availability, accessibility, and sufficiency of such guidelines is variable. In literature, nurses report a lack of the necessary knowledge to administer enteral nutrition and thus are hesitant to provide the needed care [19, 27]. In light of insufficient knowledge, availability of accessible, readable, sufficient, and clear guidelines will help to overcome barriers toward nutritional care. It will also help to reduce nurses’ noncompliant practices with the recommended evidence for enteral feeding. Educational programs are needed to promote nurses’ compliance, and such programs were found to be helpful in EN [33] and in other nursing fields among Jordanian nurses [34, 35].

In addition to the problems associated with the resources and guidelines, *unavailability of dietitians* during evening shifts, weekends, and holidays was the third barrier rated by nurses in this study. This variable was previously reported by Cahil *et al.* [25] as one of the 10 most common variables in their study. Moreover, even if the dietitians were available, it seems that they have not dedicated enough time to discuss the issues of individualized patients' problems. Nutritional support is a multidisciplinary task and the dietitian availability is vital for the delivery of safe and optimal care. Most importantly is the dietitian’s role to prepare the EN formula. Unavailability of special formulas forces nurses to hold the feeding and predisposes patients to underfeeding status. There is an individualized response of critically-ill patients to the feeding formula so management of these problems requires collaboration between nurses, physicians, and dietitians. In the absence of dietitians, the provision of EN must be delayed or stopped.

Nurses had negative attitudes regarding nutritional care since they believed that that provision of adequate nutrition did not impact patient outcomes. Nurses reported that they held feedings due to diarrhea but this fear of adverse effects was inconsistent with Cahill *et al.* [25]. In this study, nurses reported many defects in the “Guidelines Recommendations and Implementations” subscale. Such shortcomings of the guidelines and protocols make nurses uncertain about the management, consequences, and the complications of EN. Furthermore, in the “Delivery” subscale, poor communication amongst the ICU team regarding management resulted in delaying the initiation or progression of EN. Instead of discussing these fears, nurses decided to delay or stop feeding.

Based on socio-demographic variables, no significant differences were found in relation to the total score of EN barriers. Regarding the “Resources” subscale, nurses with Master degrees reported fewer barriers than nurses with a Baccalaureate degree. Similarly, nurses with the previous education of EN reported fewer barriers than nurses without previous education. It seems that nurses with upgraded knowledge better understand the importance of EN and its impact on the health of the critically-ill patients, and they did not report delayed feedings under the shortage of resources. In regards to the “Delivery” subscale, nurses in the educational hospitals reported fewer barriers than nurses from the private hospitals. Educational hospitals have students from different disciplines who participate in the care provided to the patients. In addition, they share their current knowledge and evidence-based practices with the staff. This could influence nurse’s perception of EN barriers, and overcome the delivery of EN barriers.

This study has some limitations. First, the small sample size hinders the generalizability of its results despite including nurses from different healthcare sectors in Jordan. Due to time constraints, this study did not correlate the barriers total scale and the subscales to patients’ outcomes (*e.g.*, length of ICU stay, comorbidity,…, etc.). Future studies are recommended to include a larger sample size. It is further recommended that future study correlate the barriers’ scores with patients’ outcomes, and even with nurses’ indicators, including compliance to recommended practices and satisfaction.

## CONCLUSION

This study evaluated the EN barriers perceived by Jordanian ICU nurses, and examined these barriers based on nurses’ demographics. Barriers to EN were moderately perceived with more focus on barriers regarding resources in ICU and availability of healthcare providers. Such barriers to EN are modifiable and manageable, making their identification and management crucial for optimal patient care. This study confirms that EN is a multidisciplinary responsibility and delaying this vital care will predispose patients to underfeeding and malnutrition. The impact of this scenario will be reflected in the quality of care, treatment costs, and disease process.

## Figures and Tables

**Table 1:** Description of enteral feeding barriers.

**Enteral Feeding Barriers**	**Mean(SD)**	**% of Agreement**
**Guideline Recommendations and Implementation Strategies**	4.19(1.12)	
1. I am not familiar with our current guidelines for nutrition in the ICU.	4.16(1.81)	47%
2. Current scientific evidence supporting some nutrition interventions is inadequate to inform practice.	4.39(1.64)	4%
3. The language of the recommendations of the current guidelines for nutrition is not easy to understand.	3.86(1.82)	37%
4. The current guidelines for nutrition are not readily accessible when I want to refer to them.	4.14 (1.76)	40%
5. No feeding protocol in place to guide the initiation and progression of enteral nutrition.	4.28(1.79)	45%
6. Current feeding protocol is outdated.	4.32(1.62)	44%
**ICU Resources**	4.33(1.49)	
1. Not enough nursing staff to deliver adequate nutrition.	4.80(1.81)	60%
2. Enteral formula not available on the unit.	3.90(1.98)	40%
3. No or not enough feeding pumps on the unit.	4.30(1.98)	47%
**Dietitian Support**	4.17(1.40)	
1. Waiting for the dietitian to assess the patient.	3.85(1.85)	37%
2. Not enough dietitian time dedicated to the ICU during regular weekday hours.	4.12(1.78)	44%
3. No or not enough dietitian coverage during evenings, weekends and holidays.	4.29(1.89)	51%
4. There is not enough time dedicated to education and training on how to optimally feed patients.	4.42(1.62)	53%
**Delivery of Enteral Nutrition to the Patient**	4.04(1.23)	
1. No feeding tube in place to start feeding.	3.76(1.88)	38%
2. Delay in physicians ordering the initiation of EN.	3.92(1.73)	41%
3. Waiting for physician/radiology to read x-ray and confirm tube placement.	4.00(1.81)	44%
4. Delays in initiating motility agents in patients not tolerating enteral nutrition (i.e. high gastric residual volumes).	4.08(1.49)	37%
5. Delays and difficulties in obtaining small bowel access in patients not tolerating enteral nutrition (i.e. high gastric residual volumes).	4.15(1.60)	40%
6. In resuscitated, hemodynamically stable patients, other aspects of patient care still take priority over nutrition.	4.09(1.70)	44%
7. Poor communication amongst the ICU team regarding the nutrition management resulting in delays in initiating or progression of EN.	4.31(1.67)	49%
**Critical Care Provider Attitudes and Behaviors**	4.28(1.20)	
1. Non-ICU physicians (i.e. surgeons, gastroenterologists) requesting patients not be fed enterally.	3.63(1.76)	34%
2. Nurses failing to progress feeds as per the feeding protocol.	4.05(1.51)	30%
3. Feeds being held due to diarrhea.	4.58(1.65)	57%
4. Fear of adverse events due to aggressively feeding patients.	4.59(1.50)	56%
5. Feeding being held too far in advance of procedures or operating room visits.	4.34(1.59)	50%
6. General belief among ICU team that provision of adequate nutrition does not impact on patient outcome.	4.48(1.71)	53%

**Table 2:** Significant Differences in Nurses' Perceived EN Barriers based on their Demographics.

**Demographics**	**Barriers Subscale**	**Mean(±SD)**	***P***
Healthcare Sector Private Educational	Delivery	4.43 3.50	.001
Healthcare Sector Private Educational Public	Attitudes	4.77 3.71 4.09	.000
Education Bachelor Master	ICU Resources	4.48 3.54	.007
Previous Education Yes No	ICU Resources	3.81 4.61	.003
